# Characterization of Human Mesenchymal Stem Cells Isolated from the Testis

**DOI:** 10.1155/2018/4910304

**Published:** 2018-09-03

**Authors:** Letizia De Chiara, Elvira Smeralda Famulari, Sharmila Fagoonee, Saskia K. M. van Daalen, Stefano Buttiglieri, Alberto Revelli, Emanuela Tolosano, Lorenzo Silengo, Ans M. M. van Pelt, Fiorella Altruda

**Affiliations:** ^1^Centro di Eccellenza DeNothe, Department of Biomedical, Experimental and Clinical Sciences, University of Florence, Viale Pieraccini 6, 50139 Firenze, Italy; ^2^Molecular Biotechnology Center, Department of Molecular Biotechnology and Health Sciences, University of Turin, Via Nizza 52, 10126 Turin, Italy; ^3^The Institute of Biostructure and Bioimaging (CNR) c/o Molecular Biotechnology Center, Turin, Italy; ^4^Center for Reproductive Medicine, Women's and Children's Hospital, Academic Medical Center, University of Amsterdam, Meibergdreef 9, 1105 AZ Amsterdam, Netherlands; ^5^Obstetrics and Gynecology 1U, Physiopathology of Reproduction and IVF Unit, Department of Surgical Sciences, Sant'Anna Hospital, University of Turin, Corso Spezia 60, 10126 Turin, Italy

## Abstract

Mesenchymal stem cells hold great promise for regenerative medicine as they can be easily isolated from different sources such as adipose tissue, bone marrow, and umbilical cord blood. Spontaneously arising pluripotent stem cells can be obtained in culture from murine spermatogonial stem cells (SSCs), while the pluripotency of the human counterpart remains a matter of debate. Recent gene expression profiling studies have demonstrated that embryonic stem cell- (ESC-) like cells obtained from the human testis are indeed closer to mesenchymal stem cells (MSCs) than to pluripotent stem cells. Here, we confirm that colonies derived from human testicular cultures, with our isolation protocol, are of mesenchymal origin and do not arise from spermatogonial stem cells (SSCs). The testis, thus, provides an important and accessible source of MSCs (tMSCs) that can be potentially used for nephrotoxicity testing *in vitro*. We further demonstrate, for the first time, that tMSCs are able to secrete microvesicles that could possibly be applied to the treatment of various chronic diseases, such as those affecting the kidney.

## 1. Introduction

Mesenchymal stem cells (MSCs) are multipotent stem cells first postulated by Owen et al. to be derived from bone marrow [1]. Since then, this stromal cell type has been found in a myriad of different tissues [2–4]. As MSCs are able to home to sites of inflammation and differentiate into various cell types and are immunomodulatory, they are ideal candidates for clinical applications. Unlike multipotent stem cells, SSCs are unipotent stem cells that reside on the basal membrane of the testis and can give rise only to spermatozoa. SSCs are defined by their ability to balance between self-renewal and differentiation, a feature which is supported by Sertoli and stromal cells that represent the main components of the SSC niche [5]. The study of SSCs has always been challenging as they are very limited in number [6, 7], and specific markers have not yet been identified [8].

Intriguingly, murine SSCs, under specific culture conditions, are able to spontaneously convert into pluripotent embryonic-like stem cells, known as germline cell-derived pluripotent stem cells (GPSCs) [9–12]. Recently, a number of groups have claimed that pluripotent stem cells can be derived from unipotent human SSCs [13, 14]. Nevertheless, these findings have been subsequently challenged [15], demonstrating that these “embryonic-like colonies” have an expression profile more similar to that of fibroblasts than to that of embryonic stem cells (ESCs) [16] and are, in fact, of mesenchymal origin [17, 18].

Here, we confirm that a mesenchymal population can be easily established from small human biopsies without employing any particular separation protocol. Interestingly, we demonstrate that these testicular MSCs (tMSCs) can be established from very small testis biopsies making them an attractive and novel source of MSC for cell therapy. Furthermore, we evaluate the microvesicle profile of these tMSCs derived from the human testis, which was never shown before. The therapeutic potential of extracellular vesicles is very broad, with applications including as a drug delivery route and as biomarkers for diagnosis [19, 20]. Extracellular vesicles extracted from stem cells may be used for treatment of many diseases including those affecting the kidney [21–23].

## 2. Materials and Methods

### 2.1. Culture Conditions and Isolation Protocol

tMSC colonies were derived from primary testicular cultures, starting with 1 × 10^6^ to 1 × 10^7^ cells and are based on previously published protocols [24, 25], from frozen-thawed testis material of TESE (testicular sperm extraction) pellets of 10 individuals that underwent IVF (*in vitro* fertilization). The protocol was approved by the LIVET Srl local Ethical Committee (approval date 14 March 2015). After obtaining the written informed consent, the testis material was donated for research. Testicular cells were isolated with a cocktail of enzymes, trypsin (Sigma), collagenase type I (Worthington), and hyaluronidase (Sigma), in MEM1x (Invitrogen) containing DNase (Sigma) and cultured in supplemented StemPro-34 (Invitrogen) medium as previously described for mouse testicular cells [26], with the omission of feeder cells. After their initial appearance, colonies morphologically resembling ESC colonies were picked and subcultured as described previously [27] with some modifications. Briefly, individual colonies were subcultured in ESC medium composed of DMEM-KO medium (Invitrogen), 20% ES qualified fetal calf serum (FCS, Gibco), 0.01 mM nonessential amino acid (NEAA, Gibco), 100 U/ml penicillin (Gibco), 100 *μ*g/ml streptomycin (Gibco), 0.05 mM *β*-mercaptoethanol (Sigma), a cocktail of insulin, transferrin, and sodium selenite (10 *μ*g/ml, 5.5 *μ*g/ml, and 5 ng/ml, resp.; ITS 100X, Sigma), and 25 ng/ml bFGF (basic fibroblast growth factor, Voden). Testicular somatic cells (TSCs) were isolated using the same protocol and maintained in MEM1x (Invitrogen) with 10% FCS.

### 2.2. Culture of IMCD3

IMCD3 (inner medullary collecting duct cells) were maintained in DMEM-F12 (Invitrogen) added with calf serum (Gibco), 100 U/ml penicillin (Gibco), and 100 *μ*g/ml streptomycin (Gibco).

### 2.3. Analysis of mRNA Expression

Total RNA was extracted from tMSCs at passage 6, according to the manufacturer's instruction (TRI Reagent®, Ambion). cDNA was synthesized starting from 500 ng of RNA as previously described [28]. Quantitative real-time PCR was performed using the following sets of primers: VASA (forward: 5′-CGCCAAACCCTTATGTTCAG-3′ and reverse: 5′-AAAAACTCTGCAGCCAACCTT-3′), VIMENTIN (forward: 5′-TACAGGAAGCTGCTGGAAGG-3′ and reverse: 5′-ACCAGAGGGAGTGAATCCAG-3′), CD90 (forward: 5′-AGGACGAGGGCACCTACAC-3′ and reverse: 5′-GCCCTCACTTGACCAGTT-3′), NANOG (forward: 5′-AGATGCCTCACACGGAGAC-3′ and reverse: 5′-TTTGCGACACTCTTCTCTGC-3′), SOX2 (forward: 5′-TGCTGCCTCTTTAAGACTA-3′ and reverse: 5′-CCTGGGGCTCAAACTTCTCT-3′), and OCT4 (forward: 5′-CTTCGCAAGCCCTCATTTC-3′ and reverse: 5′-GAGAAGGCGAAATCCGAAG-3′).

### 2.4. Immunofluorescence Staining

tMSCs were fixed with 4% paraformaldehyde for 10 minutes, and the primary antibody was diluted in 1% bovine serum albumin (BSA, Sigma) and incubated for 1 h at room temperature. The goat polyclonal anti-vimentin (Santa Cruz) antibody was used. Nuclei were counterstained with DAPI (4,6 diamidino-2-phenylindole, Sigma).

### 2.5. Flow Cytometry Analysis

Flow cytometry was performed using a FACSCalibur Cytometer (BD Biosciences); a phenotyping kit (MSC phenotyping kit, Miltenyi) was used to characterize the tMSCs. The following antibodies were used: anti-CD34PerCP, anti-CD45PerCP, anti-CD20PerCP, anti-CD14PerCP, anti-CD73APC, anti-CD9FITC, and anti-CD105PE (BD Pharmingen). Matched isotype controls were applied to determine background fluorescence levels.

### 2.6. Adipogenic Differentiation and Oil Red Staining

tMSCs were plated at a density of 3 × 10^5^ in a 6-well culture dish and incubated O/N in a humidified incubator. Adipogenic induction medium was prepared in DMEM-low glucose (Invitrogen) with the addition of 10% fetal bovine serum (FBS), 10 mM dexamethasone (Sigma), 10 mg/mL insulin (Sigma), 100 U/ml penicillin (Gibco), and 100 *μ*g/ml streptomycin (Gibco). Adipogenic maintenance medium was prepared as the induction medium with the exception of dexamethasone. The medium was added following the differentiation schedule: 4 days in the induction medium and 2 days in the maintenance medium (repeated 3 times).

For the red oil staining, cells were fixed for 10′ in 4% paraformaldehyde (PFA) and then incubated for 10′ in 60% isopropanol. The staining for the lipid droplets was accomplished through the incubation of cells with oil red O solution (Sigma) for 5′, and then the cells were washed with phosphate-buffered saline (PBS) and mounted. Nuclei were counterstained with hematoxylin (Bioptica).

### 2.7. Osteogenic Differentiation and Alizarin Red Staining

tMSCs were plated at a density of 1 × 10^5^ in a 6-well culture dish and incubated O/N in a humidified incubator. Cells were treated for 3 weeks with a commercial medium for osteogenic differentiation (Euroclone). For alizarin red S staining, cells were fixed for 10′ in 4% PFA and stained with the alizarin red solution (Sigma) for 30^″^ to 5′, while observing the reaction microscopically. Cells were dehydrated in acetone and mounted. Nuclei were counterstained with hematoxylin (Bioptica).

### 2.8. Alkaline Phosphatase Staining

For the alkaline phosphatase assay, cells at passage 6 were seeded onto a chamber slide (Thermo Scientific) and allowed to reach 80% confluence. They were then fixed with 4% PFA for 2′ and stained with a commercial kit, according to the manufacturer's instruction (Millipore).

### 2.9. Microvesicle (MV) Isolation from tMSCs and TSCs

MVs were isolated according to previously published protocols [21]. Briefly, MVs were obtained from supernatants of tMSCs at early passages (p6) and from testicular somatic cells as a control. tMSCs were cultured in DMEM-KO (Invitrogen), and testicular somatic cells in MEM1x (Invitrogen) both deprived of FCS for 24 h. After centrifugation at 3000 RPM for 10′ to remove debris, cell-free supernatants were centrifuged at 100,000*g* (Beckman Coulter Optima L-100K ultracentrifuge) for 2 h at 4°C. MV concentration was determined by protein quantification (Bradford assay, Bio-Rad).

### 2.10. Labelling of MVs Derived from tMSCs

50 *μ*g/ml of MVs was labelled with PKH67 dye (Sigma) according to the manufacturer's protocol. The MVs were then incubated on IMCD3 for 4 h, 12 h, and 24 h at 37°C. Following incubation, the cells were fixed, counterstained with DAPI, and analyzed by fluorescence microscopy.

### 2.11. Statistical Analysis

Values are reported as the mean ± standard error of mean. Statistical analysis was performed by using two-tailed Student's *t*-tests (^∗^ *P* < 0.05, ^∗∗^ *P* < 0.01, and ^∗∗∗^ *P* < 0.001) for the graphs comparing only two variables. For the analysis of more than two categories, the statistical significance was calculated with one-way ANOVA and Bonferroni posttest (^∗^ *P* < 0.05, ^∗∗^ *P* < 0.01, and ^∗∗∗^ *P* < 0.001). All the analyses were performed with PRISM5 (GraphPad Software Inc., La Jolla CA, USA).

## 3. Results

### 3.1. Mesenchymal Stem Cells from Testis Biopsies

**Figure 1:**
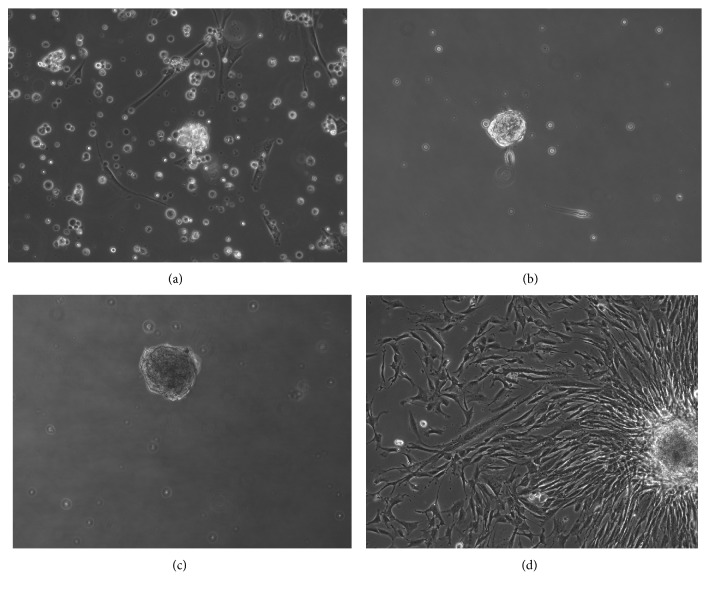
Appearance of tMSC colonies. The day after cell purification from testis biopsies, a mixed population of cells was present in the culture (a). Floating clusters started to appear between 1 week and 3 weeks postseparation (b, c). Once isolated and cultured on plastic, these colonies start to expand and proliferate (d). Original magnification: ×200.

**Figure 2:**
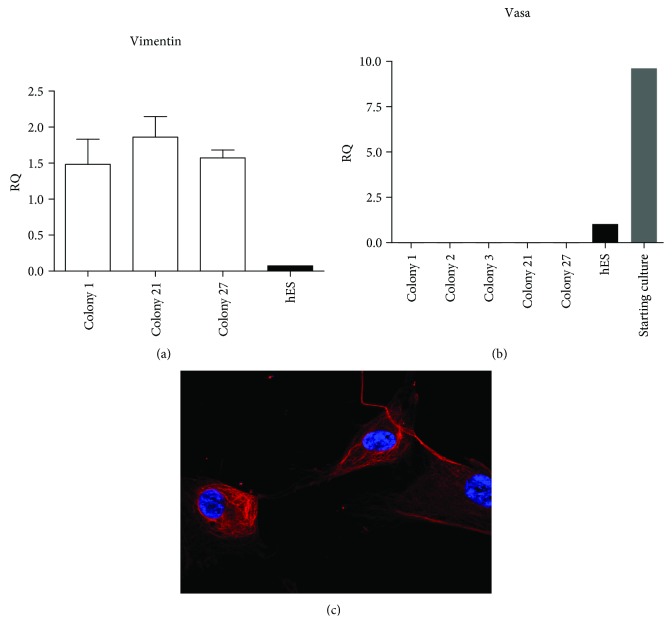
Mesenchymal phenotype of isolated tMSCs. Cells expanded from tMSC colony strongly express vimentin both at mRNA level (a) and at protein level (c). Moreover, they do not express VASA (b), a marker of cells originating from the germ lineage. Original magnification: (c) ×630.

**Figure 3:**
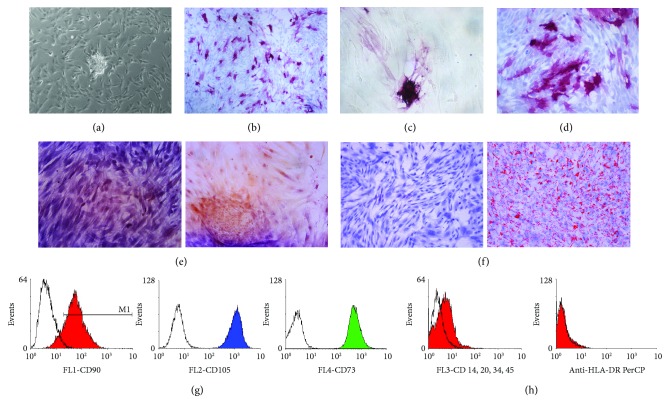
Characterization of the mesenchymal phenotype of tMSCs. (a–d) A representative picture of a tMSC colony (a) positive for AP staining and the correspondent colony in brightfield (c). Once picked and expanded, the colonies gave rise to a heterogeneous population expressing alkaline phosphatase (b–d). (e–f) Representative pictures of alizarin red staining (e) and oil red staining (f) of tMSCs, indicating osteogenic and adipogenic differentiation. Negative staining is represented on the left side, and positive staining on the right side. Original magnification: (a–c) ×100, (d) ×200, and (e, f) ×100. (g, h) A flow cytometry analysis of one of the tMSC colonies (colony #27). The analysis demonstrates that the cells are positive for CD73, CD105, and CD90 (g). As expected, the cells are negative for CD14, CD34, and HLA-DR (h). This analysis is representative of three independent experiments performed on various colonies.

Due to a lack of specific markers for SSCs [8], we chose not to sort our mixed population, composed of somatic cells, Sertoli cells, and SSCs (Sup.  Figure S1A, B, and C), isolated from testicular biopsies. One week postisolation, some cells formed aggregated floating colonies (Figure 1(a)) and became granulated with time (Figures 1(b) and 1(c)). Two days after picking, colonies attached to the plates and started to proliferate (Figure 1(d)), but despite their resemblance to ESC colonies, further analysis revealed the nongermline origin of these cells (referred to as testicular mesenchymal stem cells (tMSCs) from now on) (Figure 2). We successfully isolated 27 colonies from up to 10 different biopsies, and we were able to maintain them in culture for at least 20 passages with comparable proliferation rates. All colonies analyzed gave equivalent results and showed the same mesenchymal characteristics. RNA was extracted at different passages. The expression of VASA was not detected in any colony (Figure 2(b)). VASA is a specific protein expressed by mammalian germ cells [29], and it is essential for germ cell development [30]. Furthermore, cells derived from these colonies expressed high levels of vimentin, a typical mesenchymal marker, at both mRNA (Figure 2(a)) and protein level (Figure 2(c)). When we analyzed the expression of pluripotent markers in numerous tMSC colonies, none were found to be positive for any of the classical pluripotent markers (Sup.  Figure S2A, B, and C). Moreover, they were unable to give rise to any of the three embryonic germ layers (data not show). The ability to grow on plastic and differentiate toward three mesodermal lineages (adipo-, osteo-, and chondrogenic) and the relevant presence and absence of specific antigens are the defining criteria of MSCs [31]. These colony-derived tMSCs were capable of growing on plastic (Figure 3(a)) and were positive for CD105, CD73, and CD90 (Figure 3(g)) and negative for CD14 and CD34 (Figure 3(h)). Finally, under appropriate conditions, tMSC differentiated toward mesodermal lineages. In the case of osteogenic differentiation, calcium deposition was observed (Figure 3(e)), while lipid accumulation (Figure 3(f)) was observed in the case of adipogenic differentiation of tMSCs. Intriguingly, some of the tMSC colonies showed positive staining for alkaline phosphatase (AP) (Figure 3(c)). Once isolated and cultured, these colonies, regardless of their initial positive staining for AP, gave rise to a mixed population of cells, in which about 50% of tMSCs were positive for AP (Figures 3(b) and 3(d)), reflecting the heterogeneous nature of other types of MSCs [32]. AP is demonstrated to be identical to the mesenchymal stem cell antigen MSCA-1 [33], and it can be used as a selective marker for MSC isolation [34]. Taken together, this data confirms the mesenchymal nature of these colonies and demonstrates for the first time the expression of AP in MSCs isolated from the human testis.

### 3.2. Characterization of MVs Derived from tMSCs

**Figure 4:**
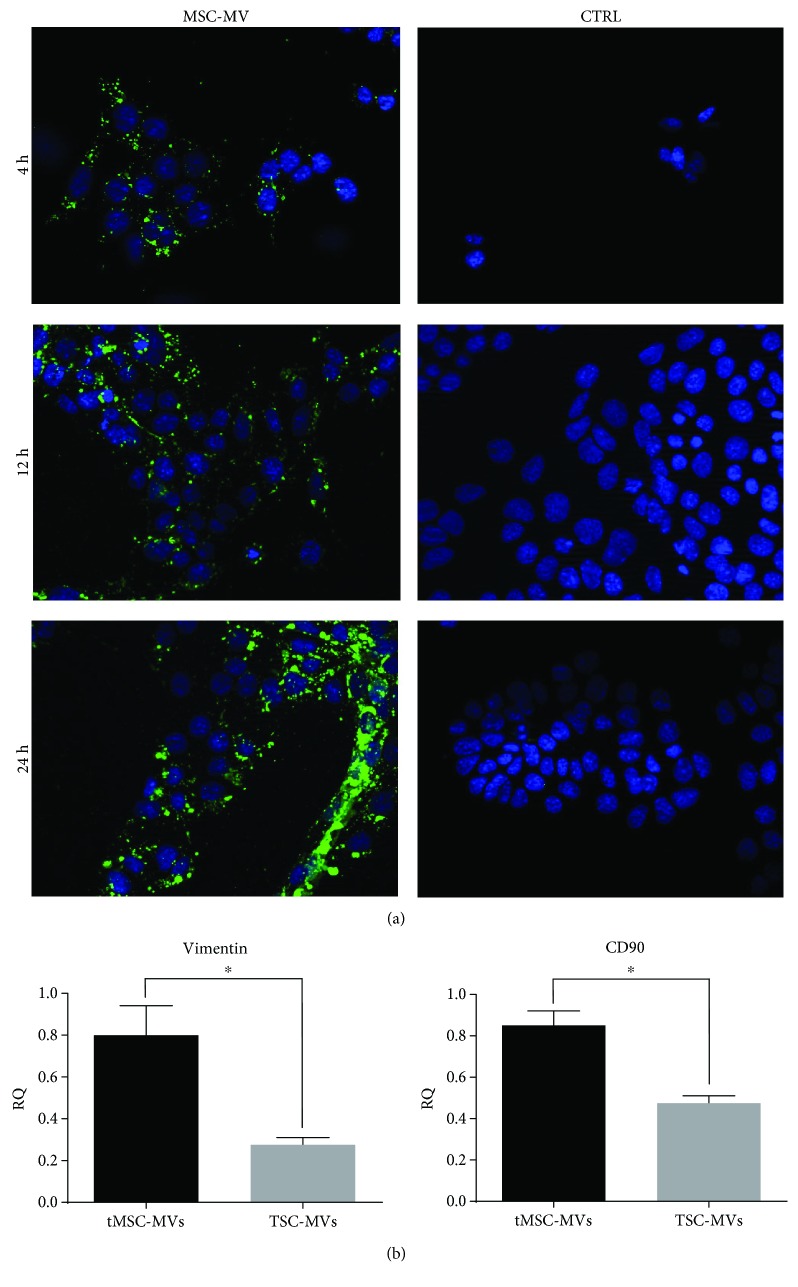
GFP^+^ MVs derived from tMSCs are incorporated by IMCD3. (a) Representative pictures of IMDC3 incubated with GFP-labelled MVs at 4, 12, and 24 hours and the control (CTRL) cells incubated only with the labelling reagent. The MVs are progressively fused with the cells as demonstrated by the increased amount of fluorescence. Original magnification: ×400. (b) Finally, real-time analysis shows that MVs derived from tMSCs are positively enriched in vimentin and CD90 when compared to the MVs obtained from testicular somatic cells isolated from the same biopsy (TSC-MVs) (*N* = 3, ^∗^ *P* < 0.05).

MVs were isolated from tMSCs following ultracentrifugation of the serum-starved culture supernatant. tMSC-derived microvesicles were labelled with PKH26 dye. IMCD3 (inner medullary collecting duct cells) were treated with the MV suspension for 4 h, 12 h, and 24 h. Uptake of MVs by the renal cells was evident at 4 h postincubation and continued in a time-dependent manner reaching the maximum uptake 24 h postincubation (Figure 4(a)). Intriguingly, we found that the testicular somatic cells used as a control were able to secrete MVs incorporated by the IMCD3, though to a lesser extent (data not shown). Real-time PCR analysis of these MVs was performed in order to evaluate their mRNA composition and verify their mesenchymal origin. We demonstrated that tMSC-MVs of mesenchymal origin express higher level of both vimentin and CD90 mRNA than MVs isolated from testicular somatic cells (Figure 4(b)). Taken together, this data demonstrates for the first time that tMSCs are able to secrete MVs that are easily taken up by renal cells.

## 4. Discussion

Mesenchymal stem cells can be obtained from a number of different tissues, including bone marrow [4], umbilical cord [2], and adipose tissue [3]. It has been previously suggested that testicular tissue contains a mesenchymal population [35] of cells, and a study from 2014 identified these mesenchymal progenitors as the source of the ESC-like colonies isolated from the human testis [17]. In the present study, we confirmed independently that the “ESC-like” colonies arising from human testicular culture are indeed of mesenchymal origin and do not possess any pluripotent characteristics. Furthermore, we demonstrated the feasibility of isolating tMSC-derived MVs, highlighting the utility of this novel source of mesenchymal stem cells as an avenue to derive MVs for regenerative medicine applications.

Different lines of tMSCs were established from up to 10 testis biopsies, all with consistent features. The isolated colonies started to appear after differential passaging around 7–14 days postisolation. Although the shape of the colonies closely resembled that of ESC colonies, once isolated, they became granulated, giving rise to a population of “spindle”-like cells able to grow on plastic as a single layer. This population of cells was positive for the expression of the mesenchymal marker vimentin at both RNA and protein levels. It is interesting to note that none of the populations arising from the various colonies showed any indication of pluripotency. When cultured in suspension, these tMSCs failed to form embryoid bodies and died afterwards (data not shown). OCT4, NANOG, and SOX2 expression was barely detectable in all of the colonies analyzed, and no expression of VASA was detected. Intriguingly, VASA expression was present in the initial mixed population isolated from testis biopsies, ruling out the possibility that the lack of VASA expression might be due to the absence of a germline population in the starting sample. When we evaluated the mesenchymal features of the testis-derived colonies, we found that not only did the cells express all the classical markers of MSCs (CD90, CD73, and CD104) but also they differentiated into cells of mesodermal origin with ease. Interestingly, we found the tMSCs to be positive for AP, another MSC marker, which was never shown before. The heterogeneous expression of AP among the cells is in line with their mesenchymal origin [36]. With a small biopsy, tMSCs are easily derived and exhibit a relatively extended life span compared to other “classical” MSCs [36], making these testicular mesenchymal stem cells amenable to expansion for therapeutic purposes, an attractive proposition for regenerative medicine.

A growing body of evidence has demonstrated that MSCs act through a paracrine effect by secreting soluble factors and microvesicles [37, 38]. MVs derived from mesenchymal stem cells can reprogram target cells suggesting that they could be exploited in regenerative medicine to repair damaged tissues, and in particular, they have been demonstrated to be efficacious in preventing acute renal injury [22, 39, 40]. MVs isolated from MSCs were successfully incorporated by IMCD3, in which the GFP staining is already present 4 h postincubation. The GFP^+^ signal increases over the time, demonstrating that the incorporation of MVs is time dependent. IMCD3 is an epithelial cell line derived from the inner medullary collecting ducts of the murine kidney; this observation may be important in light of a possible use for tMSC-MVs in the repair of renal injury. Finally, the statistically relevant expression of the two mesenchymal markers vimentin and CD90 confirms that the tMSC-MVs are definitively derived from a mesenchymal population.

## 5. Conclusions

In conclusion, we have demonstrated for the first time that mesenchymal stem cells isolated from small testis biopsies are positive for AP expression and are able to secrete MVs that can be successfully taken up by renal cells. Further analyses are required to evaluate the relevant biological activity of these new tMSC-derived MVs, with the possibility of exploiting their role and contribution in the repair of various chronic and acute diseases, as well as in nephrotoxicity testing *in vitro*.

## Supplementary Materials

Supplementary Materials Supplementary Figure 1: representative pictures of the cell types isolated from testis biopsies. Supplementary Figure 2: real-time analysis showing that tMSCs are not pluripotent.
