# Supplementary material for: Characterization of Human Mesenchymal Stem Cells Isolated from the Testis
Source: Stem Cells Int. 2018 Sep 3;2018:4910304. doi: 10.1155/2018/4910304 (PMC6140008; doi:10.1155/2018/4910304)
Supplement: Supplementary Materials — Supplementary Figure 1: representative pictures of the cell types isolated from testis biopsies. Supplementary Figure 2: real-time analysis showing that tMSCs are not pluripotent. [file 4910304.f1.docx]

***SUPPLEMENTARY INFORMATION***


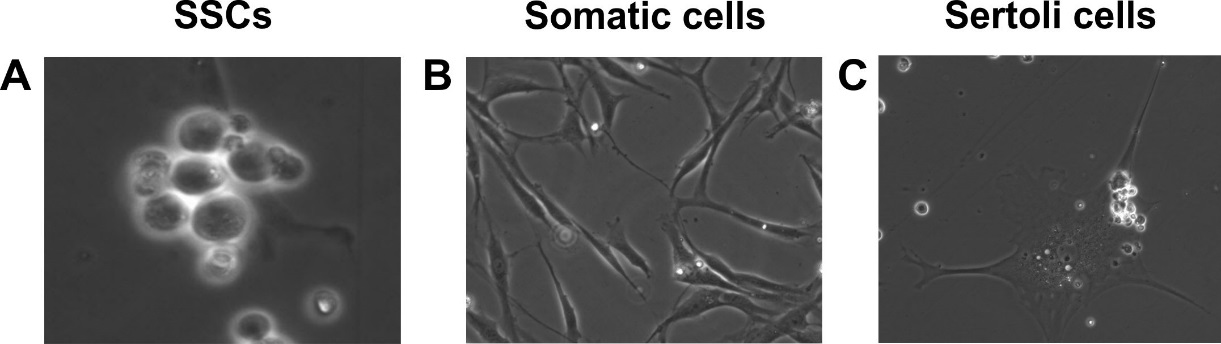


**Figure 1.** Heterogeneous composition of cells isolated from testis biopsies. Representative picture of SSCs (A), somatic cells (B) and Sertoli cell (C) at 1 week post isolation. Original magnification: x400.


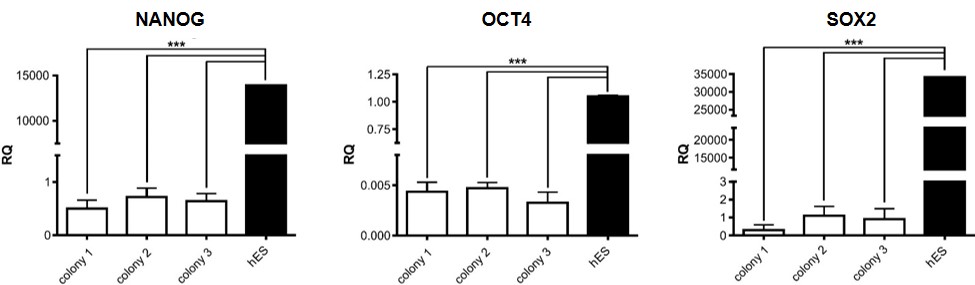


**Figure 2.** Cells derived from tMSC-colony lack expression of pluripotency markers. Real-Time analysis for NANOG (A), OCT4 (B) and SOX2 (C) shows that none of the colonies analysed expressed any of the classical pluripotency markers, confirming indeed their mesenchymal origin. (N=3, *** p<0.001).
